# Endogenous Carboxyhemoglobin Level Variation in COVID-19 and Bacterial Sepsis: A Novel Approach?

**DOI:** 10.3390/microorganisms10020305

**Published:** 2022-01-27

**Authors:** Bianca-Liana Grigorescu, Irina Săplăcan, Ioana Roxana Bordea, Marius Petrisor, Oana Coman, Claudiu Ion Puiac, Ariana Toncean, Raluca Stefania Fodor

**Affiliations:** 1 Department of Pathophysiology, University of Medicine, Pharmacology, Sciences and Technology, 540142 Targu-Mures, Romania; biancagrigorescu20@yahoo.com; 2 Department of Anesthesiology and Intensive Care, Emergency County Hospital, 540136 Targu-Mures, Romania; oana.coman45@gmail.com (O.C.); tonceanariana@yahoo.com (A.T.); 3 Department of Oral Rehabilitation, University of Medicine and Pharmacy Iuliu Hatieganu, 400012 Cluj-Napoca, Romania; 4 Department of Simulation Applied in Medicine, University of Medicine, Pharmacology, Sciences and Technology, 540142 Targu-Mures, Romania; mariuspetrisor@gmail.com; 5 Department of Intensive Care, George Emil Palade University of Medicine, Pharmacy, Sciences and Technology, 540142 Targu-Mures, Romania; claudiupuiac@gmail.com (C.I.P.); ralucasolomon@umfst.com (R.S.F.)

**Keywords:** carboxyhemoglobin, sepsis, COVID-19, liver dysfunction, lactate

## Abstract

Background: The increased production of carbon monoxide (CO) in sepsis has been proven, but the blood level variations of carboxyhemoglobin (COHb) as a potential evolutionary parameter of COVID-19 and sepsis/septic shock have yet to be determined. This study aims to evaluate the serum level variation of COHb as a potential evolutionary parameter in COVID-19 critically ill patients and in bacterial sepsis. Materials and method: A prospective and observational study was conducted on two groups of patients: the bacterial sepsis group (*n* = 52) and the COVID-19 group (*n* = 52). We followed paraclinical parameters on Day 1 (D1) and Day 5 (D5) of sepsis/ICU admission for COVID-19 patients. Results: D1 of sepsis: statistically significant positive correlations between: COHb values and serum lactate (*p* = 0.024, r = 0.316), and total bilirubin (*p* = 0.01, r = 0.359). In D5 of sepsis: a statistically significant positive correlations between: COHb values and procalcitonin (PCT) (*p* = 0.038, r = 0.402), and total bilirubin (*p* = 0.023, r = 0.319). D1 of COVID-19 group: COHb levels were statistically significantly positively correlated with C-reactive protein CRP values (*p* = 0.003, r = 0.407) and with PCT values (*p* = 0.022, r = 0.324) and statistically significantly negatively correlated with serum lactate values (*p* = 0.038, r = −0.285). Conclusion: COHb variation could provide rapid information about the outcome of bacterial sepsis/septic shock, having the advantages of a favorable cost-effectiveness ratio, and availability as a point-of-care test.

## 1. Introduction

In December 2019, a coronavirus disease epidemic was reported in Wuhan, China. The World Health Organization deemed this epidemic a serious danger to global health. COVID-19 is highly infectious and has the potential to result in catastrophic complications, most notably acute respiratory distress syndrome (ARDS) [1,2]. ARDS is defined by respiratory distress and hypoxemia, as well as the appearance of bilateral infiltrates in chest imaging [3,4].

Despite the pulmonary prevalence of COVID-19, the liver impairment identified in patients is direct hepatic cell infection. Angiotensin-converting enzyme 2 (ACE2) is the host cell receptor for severe acute respiratory syndrome coronavirus 2 (SARS-CoV-2), and the virus enters the cell through transmembrane serine protease 2 [5]. Furthermore, the spike protein plays a critical role in determining the virus’s tissue tropism and host range. SARS-CoV-2 competes with angiotensin II for the internalization of ACE2. However, the binding inhibits ACE2 action, hence reducing the enzyme’s expression in the membrane. This may contribute to the renin–angiotensin–aldosterone system imbalance. The aggressive proinflammatory response in COVID-19 is one of the most important mechanisms leading to hepatic impairment that may increase serum carboxyhemoglobin (COHb) levels [6,7].

Sepsis is defined as a potentially life-threatening condition leading to multiple organ failures caused by a dysregulated host response to bacterial aggression [8,9]. The inflammatory response is anticipated and helpful in many infections, but distinguishing the life-threatening, dysregulated response of sepsis from the usual inflammatory response of uncomplicated infection has proven difficult [10].

An important issue in sepsis is that the liver produces a large amount of carbon monoxide (CO) by oxidation of heme via the heme oxygenase-1 (HO-1) pathway. HO-1 is an enzyme induced by oxidative stress, hypoxia, cytokines, endotoxins, inflammatory mediators, and other factors. Most of the HO-1 isoforms are found in the spleen and liver [11,12].

The liver is a common site of sepsis-related injury, due to its critical roles regarding bacterial or lipopolysaccharide (LPS) clearance, lactate production/clearance, increased release of pro-inflammatory cytokines that promote distal organ dysfunction (e.g., lung injury), and increased release of anti-inflammatory cytokines [8,9,13]. In COVID-19, increased cytokine levels are related to lung injury and multi-organ failure, and a severe cytokine storm may contribute to the pathophysiology of COVID-19 [14]. Numerous mechanisms of liver injury have been hypothesized; the new coronavirus may cause significant liver injury in certain cases, most likely through immunological interactions involving intrahepatic cytotoxic T cells and Kupffer cells [15].

Alterations in liver function without structural hepatobiliary abnormalities are frequent in sepsis and are linked to infections, toxins, or cytokines. The abnormal liver function is reflected in sepsis through the inhibition of hepatocyte clearance of bilirubin (producing cholestasis) and elevated transaminase levels [13].

In the early phases of liver injury, macrophages are possible contributors to local tissue destruction and the secretion of proinflammatory cytokines. Appropriate control of the host proinflammatory response progression during the acute period of sepsis may help restore liver homeostasis and avoid sepsis-induced liver injury [16].

All endogenous CO sources are active in sepsis, i.e., the increased expression of HO-1 caused by tissue hypoxia, liver failure, oxidative stress, bacteremia, and elevated serum COHb levels [9].

Possible signs of hemolysis are present in COVID-19 acute respiratory distress syndrome (ARDS) patients. Hemolysis and epithelial alveolar cell death are caused by SARS-CoV-2 infection in the lung. Furthermore, the presence of hemoptysis or rhabdomyolysis in other COVID-19 patients may induce further cellular damage, the release of heme proteins, and heme protein accumulation [17,18].

COHb is not a pathology-specific parameter, but its variation could provide early information regarding the outcome of both bacterial and viral infection. COHb levels might be utilized to monitor the progression of sepsis and could offer important information about the clinical courses of both bacterial and viral infection. The advantages of COHb measurement include a favorable cost-effectiveness ratio; this is simple to determine and available at the patient’s bedside. Increased CO production has been demonstrated in sepsis, but the usefulness of the blood level variation of COHb as a valuable parameter of sepsis evolution remains unknown [11].

The variation of COHb as an evolutive parameter in bacterial sepsis and COVID-19 has not been investigated in any research to date. COHb levels indicate endogenous CO production, and are commonly measured by arterial blood gas analysis [11,17].

This study aims to evaluate the role of COHb variation as a potential evolutionary parameter in bacterial sepsis/septic shock and in COVID-19 critically ill patients.

## 2. Materials and Methods

Our study is a pilot, prospective, observational, and ongoing study conducted on two groups of patients: the bacterial sepsis group (*n* = 52) and the COVID-19 group (*n* = 52) hospitalized in the Anesthesia and Intensive Care Department of the Târgu Mureș Emergency Clinical County Hospital and the COVID-19 UMFST Support Unit Târgu Mureș, Mureș County, Romania.

The inclusion criteria for the bacterial sepsis group were: age between 18 and 90 years; minimum 7 days of hospitalization in the intensive care unit (ICU); diagnosis of sepsis or septic shock.

The inclusion criteria for the COVID-19 group were: age between 18 and 90 years; ICU admission for COVID-19 infection; ARDS at admission.

The exclusion criteria for both groups were: patients who did not have a confirmed infection and were not diagnosed with sepsis or septic shock; patients with pre-existing liver disease, including liver cirrhosis of various etiologies and viral or toxic hepatitis; patients with neoplastic disease; patients under immunosuppressive treatment; presence of bacterial superinfection in the COVID-19 group. A table listing the major comorbidities of COVID-19 and sepsis patients was included in supplementary materials (Tables S1 and S2).

Demographic data were obtained for patients in both groups: on Day 1 (D1) and Day 5 (D5) of hospitalization in the ICU for COVID-19 positive patients, and on D1 and D5 of sepsis in the bacterial sepsis group. Several clinical and paraclinical parameters were followed: serial bacteriological tests, blood count, biochemical tests, serum lactate, blood glucose, albumin, total protein and arterial blood gas analysis.

The Acute Physiology and Chronic Health Evaluation score (APACHE II) and Sequential Organ Failure Assessment score (SOFA) were calculated for each day. The mode of mechanical ventilation and ventilatory parameters were recorded, as was the vasoactive medication.

Nasopharyngeal swab samples were obtained from the patients and COVID-19 diagnosis was established using real-time reverse transcription–PCR (RT–PCR) analysis.

COHb was determined by an arterial puncture using a standard heparinized syringe (Stat Profile Prime Plus, Manufacturer: Nova Biomedical, Waltham, MA 02454-9141 USA, year of manufacture 2018). All the obtained data were recorded in a database.

Each study day, procalcitonin (PCT), C-reactive protein (CRP) and serum lactate were determined in both groups.

This study was conducted with the approval of the Hospitals Ethics Committee approval no 117/17.04.2019 for septic patients and no 2792/02.02.2021 for COVID-19 critically ill patients. The General Data Protection Regulation (GDPR) agreement was respected, and the obtained data were used for research purposes only.

### Statistical Analysis

The obtained data were recorded in a database and statistically analyzed using SPSS Statistics 17.0. Data series normality was tested using the Kolmogorov–Smirnov test. No normal distributions were identified in the analyses variables. Descriptive statistics are reported as median, minimum, maximum, percentiles (25th, 75th) and interquartile range (IQR). For each study group, we performed correlation analysis (Spearman correlation test) between COHb values and severity scores (APACHE II and SOFA), for PCT, CPR, partial pressure of oxygen (PaO2), serum lactate and total bilirubin levels. All statistical tests used a significance threshold of *p* = 0.05.

## 3. Results

### 3.1. Bacterial Sepsis Group

In the bacterial sepsis group, the average age of patients was 64 years (minimum age 24 years, maximum age 84 years). The gender distribution was 24 female and 28 male patients. We included both smokers and non-smokers; 35 patients were non-smokers, and 17 were smokers.

The bacteriological samples included repeated sets of blood cultures, bacteriological examinations of bronchial aspirate, urine cultures and surgical wound cultures (Table 1).

#### 3.1.1. Day 1 of Bacterial Sepsis

Descriptive statistics for PCT, CRP, serum lactate, COHb, total bilirubin, PaO2 and severity scores for D1 are displayed in Table 2.

**Figure 1:**
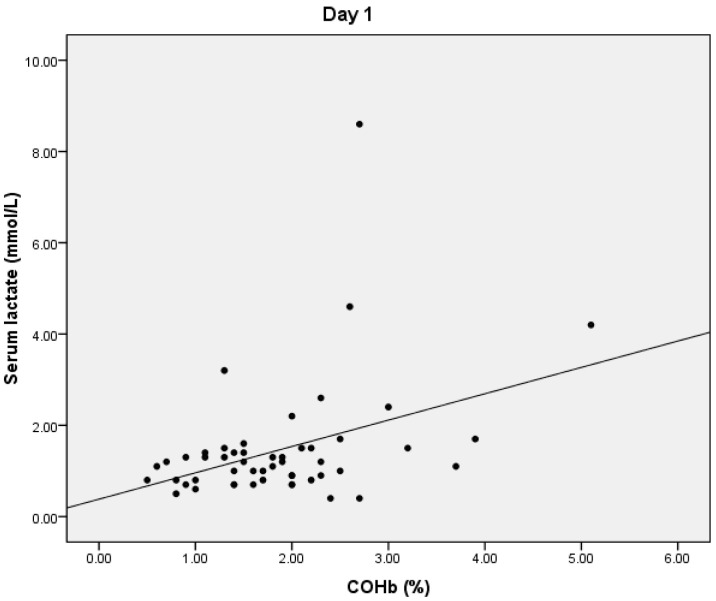
Correlation between COHb and serum lactate levels.

**Figure 2:**
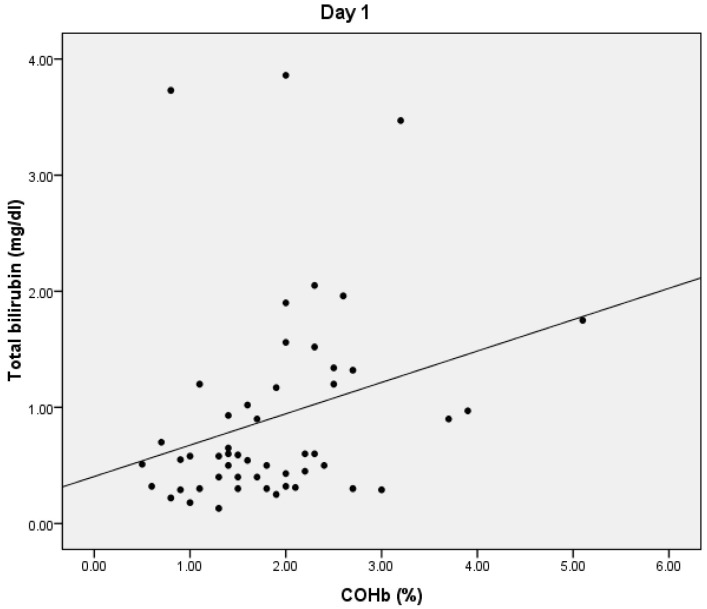
Correlation between total bilirubin and COHb.

On D1, we found a statistically significant positive correlation between serum lactate and COHb values (*p* = 0.024), with a correlation coefficient of 0.316 (Figure 1) and a significant positive correlation between total bilirubin and COHb levels (*p* = 0.01, r = 0.359) (Figure 2). We also found a significant negative correlation between COHb and PaO2 (*p* = 0.007, r = −0.376). On D1, we found no correlations between CRP, PCT, and COHb levels.

#### 3.1.2. Day 5 of Bacterial Sepsis

Descriptive statistics for PCT, CRP, serum lactate, COHb, total bilirubin, PaO2, and severity scores for D5 are shown in Table 3.

**Figure 3:**
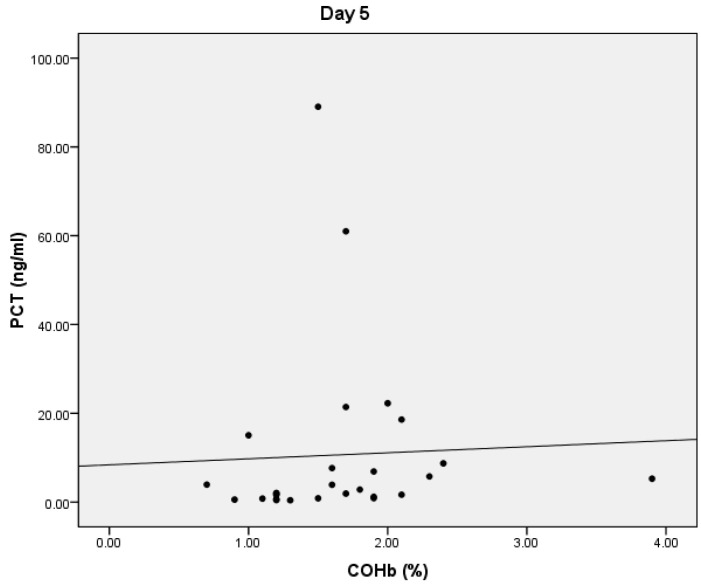
Correlation between COHb and PCT.

**Figure 4:**
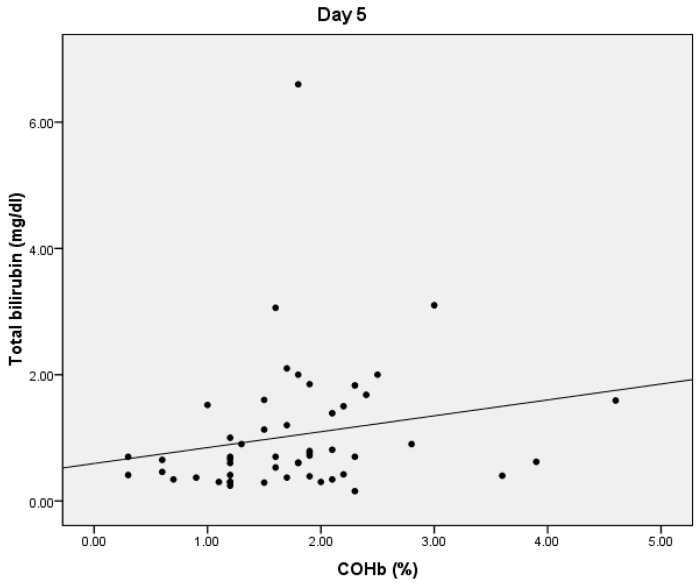
Correlation between COHb and total bilirubin.

On D5, we found statistically significant positive correlations between COHb and PCT values (*p* = 0.038, r = 0.402) (Figure 3). We also obtained a statistically significant negative correlation between the PaO2 and COHb values (*p* = 0.0001, r = −0.534) and between the total bilirubin and COHb values (*p* = 0.01, r = −0.356) (Figure 4). On D5, we found no correlations between CRP or lactate level and COHb levels.

### 3.2. COVID-19 Group

The average age in the COVID-19 group was 67 years (minimum age: 44 years, maximum age: 90 years). The gender distribution was 17 female and 36 male patients.

#### 3.2.1. Day 1 of COVID-19

Descriptive statistics for PCT, CRP, serum lactate, COHb, total bilirubin, PaO2 and severity scores for D1 are displayed in Table 4.

We found statistically significant correlations between the COHb values and CRP values (*p* = 0.003, r = 0.407) and a negative statistically significant correlation between the COHb values and serum lactate levels (*p*= 0.038, r = −0.285).

On D1, we also found a statistically significant negative correlation between serum lactate levels and PaO2 (*p* = 0.003, r = −0.396), and a significant positive correlation between lactate levels and bilirubin levels (*p* = 0.03, r = 0.288). PCT values had a statistically significant negative correlation with total bilirubin values (*p* = 0.01, r = −0.355) and a statistically significant positive correlation with COHb values (*p* = 0.022, r = 0.324). On D1, we found no correlation between COHb and total bilirubin.

#### 3.2.2. Day 5 of COVID-19

Descriptive statistics for PCT, CRP, serum lactate, COHb, total bilirubin, PaO2 and severity scores for D5 are shown in Table 5.

On D5, we found statistically significant positive correlations between the APACHE II score and PCT values (*p* = 0.001, r = 0.471), CRP values (*p* = 0.041, r = 0.290) and partial pressure of carbon dioxide (PaCO2) (*p* = 0.027, r = 0.313). We also found statistically significant positive correlations between the SOFA score and PCT values (*p* = 0.002, r = 0.428), CRP values (*p* = 0.043, r = 0.287) and PaCO2 (*p* = 0.001, r = 0.0453).

On D5, we found no significant correlations between CRP, PCT, lactate levels, and COHb levels in COVID-19 patients.

## 4. Discussion

While serial blood cultures are the “gold standard” in establishing the presence and type of infectious agent in bacterial sepsis, the most sensitive approach for detecting SARS-CoV-2 is real-time reverse transcription–PCR (RT–PCR). In bacterial sepsis, only 30–60% of patients have positive blood cultures results [19,20,21], whereas a single RT–PCR test has an 82.2% sensitivity, and if the patients are tested twice, the sensitivity rises to 90.6% [22]. Our study found positive blood cultures in 21.14% of patients, with a preponderance of gram-negative infections (61%), which are linked with an increase in liver lactate production [23].

In our study, Remdesivir and Lopinavir as antiviral treatments, and Tocilizumab as an Il-6 inhibitor, were administered to the COVID-19 group in accordance with international protocols from the time of data collection and on the recommendation of the infectious disease physician [24,25]. In the sepsis group, broad-spectrum antibiotics were administered as a first defense line using a de-escalation approach, followed by pathogen-specific antibiotic therapy based on the antibiogram, and adjusted to creatinine clearance [26].

In bacterial sepsis, a liver-mediated immune response is common and may result in liver dysfunction and alterations in endogenous COHb levels in the blood [14]. This fact was also observed in COVID-19-associated liver diseases, liver injury being related to dysregulated inflammation and related to extended hospitalization [27].

Bacterial infection triggers the hepatic metabolic pathway to produce acute-phase proteins, causing a rapid increase (hours) in CRP and PCT serum levels [28]. Candel et al. suggest that PCT can distinguish between infectious and non-infectious systemic inflammation and is considered the most sensitive biomarker to help identify bacterial sepsis due to its high sensitivity with most infections [29]. PCT has a shorter half-life than CRP and rises faster in bacterial infections [30,31]. Our study found that both COVID-19 and sepsis patients had higher median PCT values in D1 than D5. The COVID-19 group had lower PCT median levels than the sepsis group. The CRP median values decreased on D5 compared with D1, but remained within pathological limits in both groups. This could be explained by a decrease in proinflammatory response and a favorable response to treatment.

Lai et al. identified a trend indicating that PCT is superior to CRP in detecting gram-negative bloodstream infections, but the relative diagnostic ratio varies across thresholds [32]. The findings of Lai et al. and Candel et al. are consistent with ours, since the median PCT levels in the bacterial sepsis group were higher than in the COVID-19 group.

Hepatic function is essential for lactate removal because persistent hyperlactatemia or even an increase in lactate levels may indicate reduced lactate clearance rather than increased lactate production in septic patients [33,34].

In 2021, Takahashi et al. demonstrated that higher lactate clearance during the first 24 h was significantly associated with lower mortality in septic shock patients with total bilirubin levels ≥ 2 mg/dL. Lactate clearance, however, was not linked with increased mortality in individuals with total bilirubin levels less than 2 mg/dL [35]. In our study, serum lactate levels in bacterial sepsis group were increased on D5 compared with D1, most likely due to the progression of liver dysfunction, which includes decreased lactate clearance, impaired microcirculation, and hypoxia [35]. In D1 of bacterial sepsis, serum lactate values and total bilirubin values correlated positively with COHb values, because bilirubin and endogenous CO are acquired during heme catabolism through the enzyme HO-1 [12,36].

In bacterial sepsis patients, elevated COHb and bilirubin levels could be related to liver dysfunction and the disruption of the heme catabolism by increased HO-1 expression secondary to oxidative stress, hypoxia, cytokines, endotoxins, and inflammatory mediators [12]. Tissue hypoxia and impaired hepatic microcirculation in bacterial sepsis/septic shock are mechanisms synergistic with liver dysfunction and disruption of heme catabolism [37].

The supply–demand imbalance is reflected in bacterial sepsis patients by the negative correlation between COHb and PaO2, and positive correlations between COHb, serum lactate, and total bilirubin. These correlations, obtained on D1, support both the theory of hypoxia (by increasing serum lactate in correlation with COHb and decreasing PaO2 values with increasing COHb) and the theory of liver dysfunction (by COHb values increasing simultaneously with serum lactate and total bilirubin values) [36,38]. Hyperbilirubinemia is a consequence of sepsis and is used to measure liver function in SOFA scoring systems [35,39].

On D5, we found a positive correlation between COHb values and total bilirubin values that highlighted the occurrence of liver dysfunction and hypoxia in sepsis and septic shock. Impaired liver function leads to elevated bilirubin and COHb levels.

Our results are consistent with the findings of Lipinska-Gediga et al. and Takahashi et al. that at the onset of sepsis, hyperlactatemia is attributed to tissue hypoxia, and in the late stages of sepsis, lactate clearance is affected [33,37]. In this context, on D5, hyperlactatemia is not only attributed to hypoxia but also decreased lactate clearance. On D5, the same negative, statistically significant correlation was found between COHb values and PaO2 values. COHb serum level variation follows the variations of PCT and serum lactate levels on D5, and could be used as a bedside parameter for the clinical assessment of sepsis course.

Even though PaO2 mean values were in normal ranges (all the patients enrolled were mechanically ventilated), the tissue hypoxia was attributable to the impaired microcirculation that occurred in sepsis. Increased COHb levels were constantly followed by a decrease in PaO2 values. Small amounts of CO are naturally synthesized in the body, playing an important role in the regulation of physiological functions such as vasodilation, angiogenesis, vascular remodeling, protection against tissue damage, and modulation of the inflammatory response [40]. In our study, the median COHb values in the sepsis group were high (1.8% on D1 and 1. 75% on D5). The median COHb values were much lower in the COVID-19 group (0.3% in D1 and 0.4% in D5).

Melley et al. suggest that both high and low COHb levels are independently linked with mortality. Additionally, following univariate analysis, higher maximal COHb was shown to be substantially related to death [41]. Our study did not find any correlation between severity scores (APACHE II and SOFA) and estimated mortality and COHb levels. This can be attributed to the fact that we followed the variation of COHb levels, not the absolute values. In our study we attempted to use COHb variation as a metric that indicates illness progression to severe disease. COHb is not a specific parameter for bacterial/viral sepsis, therefore we cannot infer if COHb predicts survival at 30 days. To assess the influence COHb values have on patient outcome, we plotted ROC curves using in sepsis group septic shock and mortality binary variables and mortality variable in COVID-19 group. The results are attached to additional materials (Tables S3–S5, Figures S1–S3).

In the bacterial sepsis group, we included both smokers and non-smokers (17 patients were smokers and 35 were non-smokers). Boehm et al. showed that abstention from smoking for more than 12 h or smoking fewer than 20 cigarettes a day helped reduce COHb levels [42]. Given that the absolute value of COHb is higher in smokers than in non-smokers, we used the variation in COHb levels rather than its absolute values [43]. Smokers have a higher CRP serum level than non-smokers, although only slightly above the usual range. CRP values were significantly higher in our research. As a result, the increase in CRP serum level found in the present study was attributed to the inflammatory process associated with sepsis [44].

In our study, the median COHb serum level value on both study days was lower in the COVID-19 group than in the sepsis group. Acute respiratory failure, chronic obstructive pulmonary disease, pulmonary embolism, and myocardial infarction are the major causes of morbidity and death in COVID-19 and have been related to lower initial endogenous COHb levels [40,45,46].

We found a positive correlation between COHb and PCT values in both groups; however, the median COHb and PCT levels for the COVID-19 group were much lower. CRP levels decreased on D5 compared to D1, but the median levels remained within pathological limits. On D1 we found a positive correlation between CRP values and COHb values in the COVD-19 group.

The predictive significance of serum lactate and its dynamics in COVID-19 remain unknown [47]. Bruno et al. found increased mortality in COVID-19 patients with higher lactate levels, but in their study, serum lactate levels were below 2 mmol/L in the majority (68%) of patients [48]. In our study, the median value of serum lactate was below 2 mmol/L on both study days (D1: 1.6 mmol/L, D5: 1.8 mmol/L).

We observed a negative correlation between COHb value and serum lactate levels in COVID-19 patients, with median lactate values significantly higher in COVID-19 patients compared to bacterial sepsis patients on both D1 and D5, but with median values of COHb higher in bacterial sepsis on both D1 and D5.

Viral sepsis has certain similarities with bacterial sepsis, but also significant variances. Systemic inflammation affecting multiple organs is more frequent in bacterial sepsis than in COVID-19 sepsis. While bacterial sepsis is characterized by an abrupt beginning to clinical deterioration, viral diseases may have a more gradual onset and a prolonged clinical course [14,49].

The median APACHE II score on the study days was significantly higher for the COVID-19 patients than for the bacterial sepsis patients. The SOFA score was comparable between the two groups on D1 but was higher on D5 in the COVID-19 group. Zou et al. identified that the APACHE II score is a more reliable predictor of hospital mortality in patients with COVID-19 than the SOFA score. An APACHE II score equal to or more than 17 points acts as an early warning indicator of death, and may help guide additional treatment choices [50]. The median APACHE II score on D1 was 19.5 points, and the median value on D5 was 22 points.

Our current study had the following limitations: it was conducted in a single center, and a small number of patients were enrolled. This is a pilot study, and we currently have an ongoing study involving COHb serum levels in bacterial sepsis and COVID-19 infection.

COHb values can be used as an evolutionary parameter, complementary to the other biomarkers used to diagnose and monitor sepsis, but further studies are needed.

## 5. Conclusions

The variation of COHb serum levels could provide rapid, patient bedside information related to the outcome of bacterial sepsis and septic shock, but it is not useful in patients in the COVID-19 group. The advantages of COHb determination are that it has a favorable cost-effectiveness ratio, it is simple to determine, and it is available as a point-of-care test.

## Figures and Tables

**Table 1:** Bacteriological samples.

Bacteriological Samples	Gram-Negative Bacteria No of Samples (%)	Gram-Positive Bacteria No of Samples (%)	Fungal Infection No of Samples (%)
Blood cultures	6 (11.53%)	5 (9.61%)	0
Bronchial aspirate samples	32 (61.53%)	9 (17.30%)	1 (1.92%)
Urine cultures	8 (15.38%)	2 (3.84%)	0
Surgical wound cultures	5 (9.61%)	1 (1.92%)	0

**Table 2:** Values of PCT, CRP, serum lactate, COHb, total bilirubin, PaO2 and APACHE II and SOFA score—D1 of sepsis.

	Minimum	Maximum	Percentiles			IQR
			25th	50th (Median)	75th	
PCT ng/mL	0.11	208	0.55	2.28	11.93	11.38
CRP mg/dL	28.69	404.8	86.61	155	258.4	171.79
Serum lactate mmol/L	0.6	8.4	0.8	1.2	1.5	0.7
COHb%	0.5	5.1	1.3	1.8	2.3	1
Total bilirubin mg/dL	0.13	3.86	0.32	0.38	1.2	0.88
PaO2 mmHg	39	177	74	104	144	70
APACHE points	4	38	14	18	21.25	7.25
APACHE estimated mortality %	4	82	15	22	40	25
SOFA points	1	14	5	6	8.25	3.25

(CRP—C-reactive protein; PCT—procalcitonin; COHb—carboxyhemoglobin; PaO2—partial pressure of oxygen; PaCO2—partial pressure of carbon dioxide; D1—Day 1).

**Table 3:** Values of PCT, CRP, serum lactate, COHb, total bilirubin, PaO2, and APACHE II and SOFA scores—D5 of sepsis.

	Minimum	Maximum	Percentiles			IQR
			25th	50th (Median)	75th	
PCT ng/mL	0.4	89.06	0.88	2.82	8.72	7.83
CRP mg/dL	4.42	516	74.61	115.3	186.6	111.98
Serum lactate mmol/L	0.15	5	0.8	1.18	1.80	1
COHb%	0.3	4.6	1.2	1.75	2.1	0.9
Total Bilirubin mg/dL	0.15	6.60	0.40	0.70	1.51	1.11
PaO2 mmHg	39	177	76.25	106.5	143.25	67
APACHE points	5	34	12	16	23	11
APACHE estimated mortality %	3	73	15	22	40	25
SOFA score	1	17	4	6	8	4

(CRP—C-reactive protein; PCT—procalcitonin; COHb—carboxyhemoglobin; PaO2—partial pressure of oxygen; PaCO2—partial pressure of carbon dioxide; D5—Day 5).

**Table 4:** Values of PCT, CRP, serum lactate, COHb, total bilirubin, PaO2, and APACHE II and SOFA scores, D1 of COVID-19.

	Minimum	Maximum	Percentiles			IQR
			25th	50th (Median)	75th	
PCT ng/mL	0.01	79	0.09	0.33	1.16	1.08
CRP mg/dL	1.92	431	46.82	133.27	225.05	180.01
Serum lactate mmol/L	0.4	8.7	1.30	1.60	2.40	0.7
COHb%	0.1	6	0.30	0.30	0.50	0.2
Total bilirubin mg/dL	0.10	4	0.33	0.54	0.83	0.52
PaO2 mmHg	30.60	150.50	62.00	74.05	111.40	50.38
APACHE points	6	39	12.50	19.50	29.00	17
APACHE estimated mortality %	3	85	15.00	27.50	55.00	40
SOFA points	2	13	3.00	6.00	8.00	5

(CRP—C-reactive protein; PCT—procalcitonin; COHb—carboxyhemoglobin; PaO2—partial pressure of oxygen; PaCO2—partial pressure of carbon dioxide; D1—Day 1).

**Table 5:** Values of PCT, CRP, serum lactate, COHb, total bilirubin, PaO2, and APACHE II and SOFA scores—D5 of COVID-19.

	Minimum	Maximum	Percentiles			IQR
			25th	50th (Median)	75th	
PCT ng/mL	0.02	29.2	0.09	0.72	2.27	2.19
CRP mg/dL	0.79	369.83	17.32	61.46	140.90	126.53
Serum lactate mmol/L	0.6	11.7	1.40	2.10	2.70	1.3
COHb%	0.1	2.6	0.30	0.40	0.50	0.20
Total bilirubin mg/dL	0.18	5.0	0.35	0.51	0.75	0.41
PaO2 mmHg	29.9	157.3	55.95	69.10	95.30	39.5
APACHE points	4	50	15.00	22.00	34.00	19
APACHE estimated mortality %	4	85	25.00	40.00	73.00	48
SOFA points	2	15	5.00	7.00	12.00	7

(CRP—C-reactive protein; PCT—procalcitonin; COHb—carboxyhemoglobin; PaO2—partial pressure of oxygen; PaCO2—partial pressure of carbon dioxide; D5—Day 5).

## Data Availability

The data used for this study can be found in the database of the Târgu Mureş County Emergency Clinical Hospital, Mureş Romania.

## Supplementary Materials

The following supporting information can be downloaded at: https://www.mdpi.com/article/10.3390/microorganisms10020305/s1, Figure S1. ROC curve for the association between COHb and mortality in sepsis group. Figure S2. ROC curve for the association between COHb and septic shock in sepsis group.Figure S3. ROC curve for the association between for COHb and mortality in COVID-19 group. Table S1. Comorbidities of COVID-19 and sepsis patients. Table S2. Obesity class of COVID-19 and sepsis patients. Table S3. AUC statistics for COHb and mortality in sepsis group. Table S4. AUC statistics for COHb and septic shock in sepsis group. Table S5. AUC statistics for COHb and mortality in COVID-19 group.
