# Supplementary material for: Endogenous Carboxyhemoglobin Level Variation in COVID-19 and Bacterial Sepsis: A Novel Approach?
Source: Microorganisms. 2022 Jan 27;10(2):305. doi: 10.3390/microorganisms10020305 (PMC8878399; doi:10.3390/microorganisms10020305)
Supplement: Supplementary file 1 [file microorganisms-10-00305-s001.zip › microorganisms-1538696-supplementary.pdf]

1. A table listing the major comorbidities of COVID-19 and sepsis patients was included.

Table S1. Comorbidities of COVID-19 and sepsis patients.

	**Comorbidities (Number of patients)**							
	Without comorbidities	Cardiovascular disease	Pre-existing lung disease	Gastroenterology	Metabolic disease	Neurology	Nephrology	Cardio circulatory arrest
**COVID-19 Group**	3	48	2	6	41	5	17	4
**Sepsis Group**	0	9	11	5	40	5	1	6

Table S2. Obesity class of COVID-19 and sepsis patients.

**Obesity class (number of patients)**					
	Normostenic patients	Overweight patients	I	II	III
**Sepsis Group**	20	7	3	14	8
**COVID-19 Group**	11	7	1	14	19

2. In this research, we attempted to use COHb variation as a metric that indicates illness progression to severe disease, a parameter that is determined daily by the blood gas analysis necessary for ICU patients. COHb is not a specific parameter for bacterial/viral sepsis, therefore we cannot infer if COHb predicts survival at 30 days at this time. Our focus was on COHb as an evolutionary parameter; nevertheless, larger studies with a larger number of patients are needed to evaluate the COHb predictive value.

To assess the influence COHb values have on patient outcome, we plotted ROC curves using in sepsis group septic shock and mortality binary variables and mortality variable in COVID-19 group. All tests were two-tailed tests. All statistical tests use a 0.05 significance threshold. The only variables in the database on which ROC curves could be generated were mortality and septic shock. The aim of the study was not to follow COHb as a predictor of mortality, we propose that this extra statistic be included in the Supplementary Materials section.

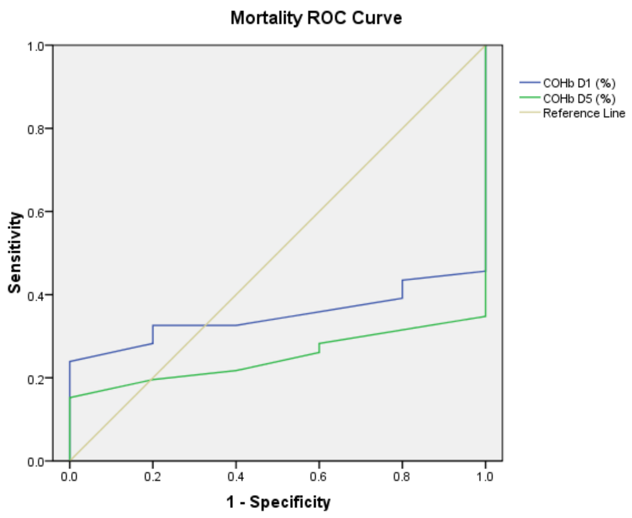

Figure S1. ROC curve for the association between COHb and mortality in sepsis group.

Table S3. AUC statistics for COHb and mortality in sepsis group.

**Area Under the Curve**					
Test Result Variable(s)	Area	Std. Error	Asymptotic Sig.	Asymptotic 95% Confidence Interval	
				Lower Bound	Upper Bound
COHb D1 (%)	0.350	0.071	0.274	0.210	0.490
COHb D5 (%)	0.250	0.065	0.069	0.123	0.377

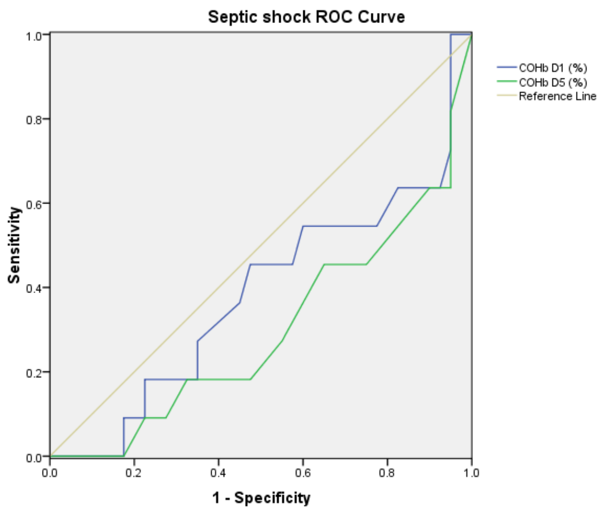

Figure S2. ROC curve for the association between COHb and septic shock in sepsis group.

Table S4. AUC statistics for COHb and septic shock in sepsis group.

Area Under the Curve					
Test Result Variable(s)	Area	Std. Error	Asymptotic Sig.	Asymptotic 95% Confidence Interval	
				Lower Bound	Upper Bound
COHb D1 (%)	0.536	0.090	0.695	0.359	0.713
COHb D5 (%)	0.590	0.090	0.322	0.413	0.767

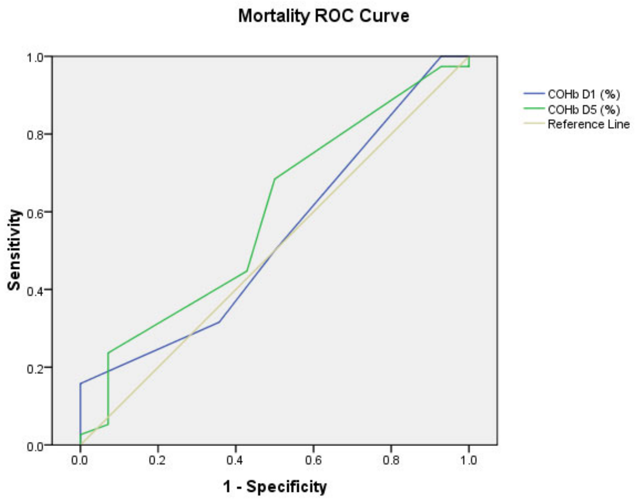

Figure S3. ROC curve for the association between for COHb and mortality in COVID-19 group.

Table S5. AUC statistics for COHb and mortality in COVID-19 group.

Area Under the Curve					
Test Result Variable(s)	Area	Std. Error	Asymptotic Sig.	Asymptotic 95% Confidence Interval	
				Lower Bound	Upper Bound
COHb D1 (%)	0.383	0.100	0.238	0.188	0.578
COHb D5 (%)	0.299	0.090	0.043	0.122	0.476

In both groups we had a mortality over 75% at 30 days. In the case of the sepsis group, this was due to both sepsis and the patient's underlying pathology. The study included patients with severe pathologies (massive strokes, polytrauma, patients with major abdominal and cerebral surgery, COPD exacerbation), complicated by sepsis. The higher mortality in the COVID-19 group was attributed to the associated comorbidities, the severe form of ARDS that patients had at admission, as well as the absence of a prevention strategy - at the time of data collection there was no vaccine for COVID-19.
